# Examining Racial and Ethnic Disparities Among Older Adults in Long-Term Care Facilities

**DOI:** 10.1093/geroni/igaa057.089

**Published:** 2020-12-16

**Authors:** Kathy Lee, Rebecca Mauldin, John Connolly, Weizhou Tang

**Affiliations:** 1 University of Texas at Arlington, Arlington, Texas, United States; 2 University of Southern California, Los Angeles, California, United States

## Abstract

Background: Nearly half-million older adults from minority racial and ethnic groups in long term care face disparities in quality of life and quality of care. However, there is little information about the associations between a resident’s race/ethnicity and the types of official complaints lodged. Methods: This project was a mixed methods study using a sequential explanatory design to examine ethnic and racial differences in types of complaints and rates of complaint resolution in a local Ombudsman Program. First, resident race/ethnicity and complaint data were collected from the Ombudsman Program and analyzed. Then, we conducted focus groups with Ombudsman Program staff and volunteers to provide a more complete interpretation of findings from the first phase. Results: Residents from ethnic/racial minority groups were less likely to generate Resident Care complaints and more likely to generate Resident Rights complaints, compared to non-Hispanic White residents (p<.05). Resident Rights, Quality of Life, and Administrative complaints were less likely to be disposed satisfactorily, compared to Resident Care complaints (p<.05). Themes emerged from our qualitative findings include language barriers and more efforts required for residents’ rights due to concerns raised more frequently among minority residents. Implications: Cultural competence training for Ombudsmen as well as care professionals should focus on skills and knowledge that value diversity, understand and respond to their unique concerns. Ombudsmen play an important role as they create an avenue for the residents to discuss their concerns. Implementation research may improve our understanding of the development and delivery of the Ombudsman Program.

